# Ideal Cardiovascular Health Metrics on the Prevalence of Asymptomatic Intracranial Artery Stenosis: A Cross-Sectional Study

**DOI:** 10.1371/journal.pone.0058923

**Published:** 2013-03-12

**Authors:** Qian Zhang, Shufeng Zhang, Chunxue Wang, Xiang Gao, Yong Zhou, Heng Zhou, Anxin Wang, Jianwei Wu, Liheng Bian, Shouling Wu, Xingquan Zhao

**Affiliations:** 1 Department of Neurology, Beijing Tiantan Hospital, Capital Medical University, Beijing, China; 2 Department of Cell Transplantation, the General Hospital of Chinese People’s Armed Police Forces, Beijing, China; 3 Department of Neurology, the General Hospital of Chinese People’s Armed Police Forces, Beijing, China; 4 Channing Laboratory, Department of Medicine, Brigham and Women’s Hospital, and Harvard Medical School, Boston, Massachusetts, United States of America; 5 Department of Nutrition, Harvard University School of Public Health, Boston, Massachusetts, United States of America; 6 Department of Cardiology, Kailuan Hospital, Tangshan, China; University of Groningen, The Netherlands

## Abstract

**Background and Purpose:**

Intracranial Artery Stenosis (ICAS) is one of the most common causes of ischemic stroke in Asia. Previous studies have shown the number of ideal cardiovascular health (CVH) metrics was associated with lower risk of stroke. This study aimed to investigate the relationship between ideal CVH metrics and prevalence of ICAS.

**Methods:**

A random sample of 5,412 participants (selected from Kailuan Study as a reference population) aged 40 years or older (40.10% women), free of stroke, transient ischemic attack, and coronary disease, were enrolled in the Asymptomatic Polyvascular Abnormalities Community study from 2010 to 2011. We collected information on the seven CVH metrics (including smoking, body mass index, dietary intake, physical activity, blood pressure, total cholesterol and fasting blood glucose); and assessed ICAS by transcranial Doppler. The relationship between the ideal CVH metrics and prevalence of ICAS was analyzed using the multivariate logistic regression.

**Results:**

After adjusting for age, sex, and other potential confounders, the adjusted odds ratios(95% confidence interval) for ICAS were 0.76(0.58–0.99), 0.55(0.43–0.72), 0.49(0.37–0.65), 0.43(0.31–0.61), and 0.36(0.22–0.62), respectively, for those having 2, 3, 4, 5, and 6–7 ideal CVH metrics compared with those having 0–1 ideal metric(p-trend<0.0001). Similar inverse associations were observed in different age and gender groups (all p-trends<0.05).

**Conclusion:**

We found a clear gradient relationship between the number of ideal CVH metrics and lower prevalence of ICAS in a Chinese population, which supports the importance of ideal health behaviors and factors in the prevention of ICAS.

## Introduction

Intracranial arterial stenosis (ICAS) is one of the most common causes of stroke [1], [2], [3], which accounts for 8–10% of all cerebral ischemic events every year [4], [5], [6], [7], and this proportion is higher in Asia. ICAS was found in 33–37% of Chinese patients with ischemic stroke [8], [9] and 51% of those with transient ischemic attack [10]. The reasons for such racial difference in distribution of intracranial and extracranial atherosclerosis remain uncertain [11]. Genetic susceptibility may play a key role and there may be different pathophysiologies for the two locations [12], [13]. Other possible reasons include different prevalence of risk factors or certain life styles across races [7]. Given the high prevalence of ICAS in Asia, studying risk factors or protective factors for ICAS in Asian populations will have greater public health and clinical implications. Developing efficient preventive approaches for ICAS may be an effective strategy to minimize the risk of stroke and health burden among general populations. Early detection may lead to therapeutic intervention while patients are still asymptomatic [14]. Recently, the American Heart Association (AHA) defined the concept of “ideal cardiovascular health(CVH)” metrics that included four ideal health behaviors (nonsmoking, normal weight, physical activity at goal levels, and a healthy diet) and three ideal health factors (normal cholesterol, blood pressure, and fasting blood glucose) [15]. The underlying concept of the AHA ideal CVH is “primordial prevention”, which is distinctly different from “primary prevention” and designed to practice healthier behaviors, preventing the emergence of risk factors, rather than disease prevention [16]. In our previous study based on 2,308 Chinese adults, we found a significant positive association between the Framingham stroke risk profile (FSRP) and risk of having ICAS [17]. However, the FSRP, which was based on the concept of “primary prevention”, does not include several important health behavior components, such as normal weight and physical activity. In contrast, several clinical indicators, such as atrial fibrillation, left ventricular hypertrophy, and history of cardiovascular disease, were included in the FSRP. In this context, it is of interest to examine the association between the AHA CVH and risk of ICAS, which could improve our understanding regarding the potential implication of “primordial prevention” strategy in the prevention for ICAS. We thus conducted a cross-sectional analysis to examine whether adherence to the AHA CVH metrics was associated with a lower likelihood of having ICAS among 5,412 Chinese adults who participated in an ongoing Chinese cohort.

## Methods

### Study Design and Population

The Asymptomatic Polyvascular Abnormalities Community study (APAC) is a community-based, observational study, to investigate the epidemiology of asymptomatic ICAS, carotid atherosclerosis and peripheral artery diseases in Chinese adults. A total of 5,852 subjects were randomly sampled from our reference population of 101,510 participants (81,110 males and 20,400 females, aged 18–98 years old) in Kailuan study [18], which was an ongoing prospective study from 2006 conducted in Kailuan community in Tangshan city, a large and littoral modern city located in the southeast of Beijing. The inclusion criteria included: (1) aged 40 years or older; and (2) free of stroke, transient ischemic attack, and coronary disease. From June 2010 to June 2011, a total of 5,440 participants who provided the informed consent and completed the baseline survey were eligible and recruited in the APAC study. During the baseline survey, all the participants had undergone questionnaire assessment, clinical, laboratory, and transcranial Doppler (TCD) examinations. We excluded 28 participants who had incomplete data on health factors or behaviors, leaving 3,242 men and 2,170 women in the analyses. The APAC study was performed according to the guidelines of Helsinki Declaration and was approved by both the Ethics Committees of the Kailuan General Hospital and Beijing Tiantan Hospital. Written informed consent was obtained from all participants and approved by the above ethics committees.

### Assessment of Cardiovascular Health Metrics

Information on smoking, dietary intake, and physical activity was collected via questionnaires. Smoking status was classified as “never”, “former”, or “current” according to self-reported information. Dietary data, mainly salt intake, were classified into three categories “low”, “medium”, or “high”, with a definition of <6 gram/day, 6–10 gram/day, or >10 gram/day, respectively, which was roughly estimated according to the standard salt spoon. Physical activity was classified into three groups “very active”, “moderately active”, or “inactive”, with a definition of more than 80 minutes per week, less than 80 minutes per week, or none, respectively. Based on participants’ response to each relevant question, we further categorized smoking status, dietary intake, and physical activity into the corresponding three groups: “ideal”, “intermediate”, and “poor” respectively. For example, we considered non-smokers into an “ideal” group, former smokers into an “intermediate” group, and current smokers into a “poor” group.

We measured weight (to the nearest 0.1 kg) and height (to the nearest 0.1 cm), and calculated body mass index (BMI) as body weight (kg) divided by the square of height (m^2^). Blood pressure was measured to the nearest 2 mm Hg using a mercury sphygmomanometer with a cuff of appropriate size. Two readings of systolic blood pressure (SBP) and diastolic blood pressure (DBP) were taken at a five-minute interval after participants had a rest in a chair for at least five minutes. The average of the two readings was used for the current data analysis. If the two measurements differed by more than 5 mm Hg, an additional reading was taken, and the average of the three readings was used. Hypertension was defined as presence of a history of hypertension, or using antihypertensive medication, or a SBP≥140 mm Hg, or a DBP≥90 mm Hg.

Blood samples were collected from the antecubital vein in the morning after an overnight fast (>8 hr) and transfused into vacuum tubes containing EDTA (Ethylene Diamine Tetraacetic Acid). Fasting blood glucose was measured with the hexokinase/glucose-6-phosphate dehydrogenase method. Cholesterol and triglyceride were measured enzymatically (interassay coefficient of variation: <10%; Mind Bioengineering Co. Ltd, Shanghai, China). The other blood samples were measured using an auto-analyzer (Hitachi 747; Hitachi, Tokyo, Japan) at the central laboratory of the Kailuan General Hospital. Diabetes mellitus was defined as a self-reported history, currently treated with insulin or oral hypoglycemic agents, or fasting blood glucose level ≥126 mg/dl. Hyperlipidemia was defined as a self-reported history, current use of cholesterol lowering medicine, or total cholesterol level ≥220 mg/dl or triglyceride ≥150 mg/dl.

According to the AHA definitions [15], BMI was classified as ideal (<25 kg/m^2^), intermediate (25 to 29.9 kg/m^2^) or poor (≥30 kg/m^2^); blood pressure was classified as ideal (SBP<120 mm Hg and DBP<80 mm Hg and untreated), intermediate (120 mm Hg≤SBP≤139 mm Hg, 80 mm Hg≤ DBP≤89 mm Hg, or treated to SBP/DBP<120/80 mm Hg), or poor (SBP≥140 mm Hg, DBP≥90 mm Hg, or treated to SBP/DBP>120/80 mm Hg); Fasting blood glucose was classified as ideal (<100 mg/dL and untreated), intermediate (100 to 125 mg/dL or treated to <100 mg/dL), or poor (≥126 mg/dL or treated to ≥100 mg/dL); and total cholesterol status was classified as ideal (<200 mg/dL and untreated), intermediate (200 to 239 mg/dL or treated to <200 mg/dL), or poor (≥240 mg/dL or treated to ≥200 mg/dL), respectively.

To examine the overall effects of these health metrics, we created a dichotomized variable for each component of the health metrics: “ideal” was coded as 1 and “non-ideal” (combined “intermediate” and “poor” categories) was coded as 0.

### Assessment of Potential Covariates

Information on demographic variables (e.g., age, sex, household income, education and family history of stroke) was collected via questionnaires. The participants’ age was classified into two categories: 40–59 years old and ≥60 years old. The average monthly income of every family member was reported as “<¥1,000”, “¥1,000–3,000”or “≥¥3,000”. The education level was categorized as “high school or higher”, “middle school” or “illiteracy or primary school”.

### Assessment of ICAS

TCD was performed by two experienced neurologists using portable machines (Nicolet/EME Company, Germany). The two neurologists were blinded to the baseline information of the participants. ICAS was diagnosed according to the peak systolic flow velocity in terms of Wong’s published criterion [19]. The stenotic arteries were defined as: >140 cm per second for the middle cerebral artery, >120 cm per second for the anterior cerebral artery, >100 cm per second for the posterior cerebral artery and vertebra-basilar artery, and >120 cm per second for the siphon internal carotid artery. In addition to the above velocity criteria, we took into account patients’ age, the presence of turbulence or musical sound, and whether the abnormal velocity was segmental. Subjects without good temporal windows were considered non-stenotic.

### Statistical Analyses

Statistical analyses were performed using SAS software, version 9.1 (SAS Institute, Cary, North Carolina, USA). Continuous variables were described by mean (standard deviation [SD]) and compared using an ANOVA analysis. Categorical variables were described by percentages and compared using Chi Square tests. Logistic regression was used to estimate the prevalence of ICAS across health metric categories by calculating the odds ratios (ORs) and 95% confidence interval (CI). Because only few participants were in the group of 0 or 7 ideal CVH metrics, we combined the group of 0/1 ideal metric and the group of 6/7 ideal metrics together, respectively. We adjusted for age, sex, education, income and family history of stroke in the models because they are known or possible risk factors for ICAS in previous studies [19], [20], [21], [22], [23], and were associated with the exposure (i.e., the ideal health metrics) as shown in Table 1. All statistical tests were 2-sided, and a significant level was set as 0.05.

**Table 1:** Basic Characteristics of Participants regarding the Number of Ideal Cardiovascular Health Metrics.

	Number of ideal cardiovascular health metrics							
	0	1	2	3	4	5	6 or 7	P value
Total(%,n)	2.18(118)	9.98(540)	21.03(1138)	26.42(1430)	23.19(1255)	12.92(699)	4.29(232)	
Women (%,n)	0.85(1)	17.41(94)	25.31(288)	38.60(552)	50.60 (635)	62.37(436)	70.69(164)	<0.0001
Mean age ± SD (y)	52.39±9.51	54.24±10.49	55.21±11.04	55.60±11.54	56.08±12.62	54.61±12.72	52.91±12.73	<0.0001
Education (%,n)								
Illiteracy/Primary	18.64(22)	15.56(84)	13.27(151)	13.36(191)	10.53(132)	9.44(66)	5.60(13)	<0.0001
Middle school	40.68(48)	48.89 (264)	48.59(553)	46.29(662)	42.82(537)	34.19(239)	34.48(80)	
High school or above	40.68(48)	35.56(192)	38.14(434)	40.35 (577)	46.65(585)	56.37(394)	59.91(139)	
Income (%,n)*								
<¥1,000	24.58(29)	19.44(105)	22.32(254)	21.96(314)	20.89(262)	20.20(141)	17.67(41)	0.0287
¥1,000–3,000	66.10(78)	71.30(385)	66.34(755)	65.87(942)	64.27(806)	65.04(454)	67.67(157)	
≥¥3,000	9.32(11)	9.26(50)	11.34(129)	12.17(174)	14.83(186)	14.76(103)	14.66(34)	
Previous history of disease								
Diabetes (%,n)	33.05(39)	26.48(143)	18.28(208)	10.49(150)	7.73(97)	1.86(13)	0.86(2)	<0.0001
Hypertension (%,n)	64.41(76)	67.22(363)	61.60(701)	52.38(749)	40.64(510)	24.03(168)	13.36(31)	<0.0001
Dyslipidemia (%,n)	88.98(105)	78.52(424)	61.07(695)	50.21(718)	36.97(464)	25.75(180)	21.12(49)	<0.0001
Family history of stroke (%,n)	17.80(21)	19.07 (103)	16.78(191)	18.53(265)	18.25(229)	16.31(114)	21.55(50)	0.5145

Note: *Average monthly income of every family member.

## Results

We identified 711 ICAS (13.14%) among 3,242 men and 2,170 women, with a prevalence of 12.07% and 13.85%, respectively.

Table 1 shows basic characteristics of participants regarding the number of ideal CVH metrics. Those with a relatively larger number of ideal CVH metrics were more likely to be women, younger, and had higher education and income. We did not observe any significant differences on family history of stroke between different numbers of ideal CVH metrics.

Table 2 shows the relationship between each component of the CVH metrics and the prevalence of ICAS. After adjusting for sex, age, education, income, family history of stroke, and the other six metrics, we found the ideal blood pressure or fasting blood glucose metric was significantly associated with the low prevalence of ICAS in any sex or age group, when compared with the non-ideal group, respectively. The ideal total cholesterol was significantly associated with the lower prevalence of ICAS in both age groups, but only significant for male, not for female. We also found a significant association between ideal smoking status and the lower prevalence of ICAS in the total population, however, in further subgroup analyses, only male and elder groups(≥60 years of age) reached a significant level, respectively. No significant relationship was found between BMI, diet, or physical activity and the prevalence of ICAS in any group. We also calculated the changes in the area under the receiver-operating curve (AROC) by gradually adding each individual CVH component based on OR values into the model. The AROC increased from 0.656 to 0.691.

**Table 2:** Odds Ratios (ORs) with 95% CI of Ideal to Non-ideal Group of Each Cardiovascular Health Metric for ICAS*.

Metrics	Total	Gender		Age	
		Male	Female	40–59 y	≥60 y
Smoking	0.74(0.60–0.92)	0.64(0.51–0.81)	1.08 (0.44–2.65)	0.76(0.54–1.06)	0.63(0.48–0.84)
P value	0.0057	0.0002	0.8589	0.1024	0.0015
BMI (kg/m2)	1.02(0.86–1.21)	0.88(0.71–1.09)	1.23(0.93–1.61)	1.02(0.82–1.27)	1.01(0.78–1.31)
P value	0.8038	0.2369	0.1415	0.8724	0.9242
Physical activity	1.01(0.85–1.20)	0.98(0.78–1.22)	1.03(0.78–1.36)	1.07(0.84–1.36)	0.90(0.70–1.16)
P value	0.9318	0.8335	0.8504	0.5924	0.4088
Diet	1.21(0.998–1.48)	1.27(0.98–1.65)	1.20(0.89–1.62)	1.24(0.95–1.61)	1.25(0.93–1.69)
P value	0.0522	0.0737	0.2353	0.1105	0.1446
Total cholesterol	0.75(0.63–0.88)	0.60(0.49–0.75)	0.93(0.71–1.22)	0.73(0.58–0.91)	0.70(0.54–0.91)
P value	0.0006	<0.0001	0.6035	0.0054	0.0063
Blood pressure	0.50(0.37–0.67)	0.33(0.19–0.56)	0.50(0.34–0.72)	0.51(0.36–0.71)	0.33(0.17–0.62)
P value	<0.0001	<0.0001	0.0002	<0.0001	0.0007
Fasting blood glucose	0.62(0.53–0.74)	0.60(0.49–0.75)	0.61(0.46–0.80)	0.67(0.53–0.84)	0.54(0.42–0.70)
P value	<0.0001	<0.0001	0.0004	0.0006	<0.0001

CI: confidence interval; ICAS: intracranial artery stenosis; BMI: body mass index.

* The following potential confounders were adjusted for each OR: sex, age (year), education, average monthly income of the family members, family history of stroke, and the other six cardiovascular health metrics.

The ORs of ICAS by the number of ideal CVH metrics are shown in Table 3. The crude ORs of prevalence of ICAS in the cohort were lower among those with a greater number of ideal CVH metrics. After adjusting for age, sex, education, income and family history of stroke, the similar pattern still existed (p-trend<0.0001). The significant inverse associations were found in either age group (p-trend<0.0001), both male (p-trend<0.0001) and female (p-trend = 0.0209).

**Table 3:** Odds Ratios (ORs) for ICAS by the Number of Ideal Cardiovascular Health Metrics.

	Number of ideal cardiovascular health metrics						
	0&1	2	3	4	5	6&7	P trend
Total							
Participants (n)	658	1138	1430	1255	699	232	
Cases (n)	118	176	179	148	71	19	
Crude OR(95% CI)	1	0.84(0.65–1.08)	0.66(0.51–0.84)	0.61(0.47–0.80)	0.52(0.38–0.71)	0.41(0.25–0.68)	<0.0001
Model 1(95% CI)*	1	0.75(0.58–0.98)	0.55(0.42–0.71)	0.48(0.36–0.63)	0.42(0.30–0.58)	0.35(0.21–0.59)	<0.0001
Model 2(95% CI)†	1	0.76(0.58–0.99)	0.55(0.43–0.72)	0.49(0.37–0.65)	0.43(0.31–0.61)	0.36(0.22–0.62)	<0.0001
<60(year)							
Participants (n)	507	828	1011	863	513	183	
Cases (n)	66	85	89	74	40	12	
OR(95% CI)‡	1	0.71(0.50–1.00)	0.55(0.39–0.78)	0.50(0.34–0.73)	0.45(0.29–0.70)	0.37(0.19–0.72)	<0.0001
≧60(year)							
Participants (n)	151	310	419	392	186	49	
Cases (n)	52	91	90	74	31	7	
OR(95% CI)‡	1	0.78(0.51–1.19)	0.50(0.33–0.77)	0.42(0.27–0.64)	0.34 (0.20–0.58)	0.27(0.11–0.65)	<0.0001
Men							
Participants (n)	563	850	878	620	263	68	
Cases (n)	98	136	109	72	27	7	
OR(95% CI)§	1	0.83(0.61–1.11)	0.52(0.38–0.71)	0.38(0.27–0.55)	0.30(0.19–0.49)	0.25(0.11–0.58)	<0.0001
Women							
Participants (n)	95	288	552	635	436	164	
Cases (n)	20	40	70	76	44	12	
OR(95% CI)§	1	0.59 (0.32–1.08)	0.56(0.32–0.99)	0.56(0.32–0.97)	0.48(0.27–0.88)	0.36(0.17–0.79)	0.0209

* Model 1: Adjusted for sex and age (year).

† Model 2: Adjusted for sex, age (year), education, average monthly income of every family member, and family history of stroke.

‡ Adjusted for sex, education, average monthly income of every family member, and family history of stroke.

§ Adjusted for age (year), education, average monthly income of every family member, and family history of stroke.

## Discussion

In the current study, we found that individuals who met six or more ideal CVH metrics were 64% less likely to have ICAS, relative to those who met only one metric or did not meet any metrics. Similar inverse associations were observed in either sex or age subgroup. To the best of our knowledge, this is the first study to demonstrate the relationship between the AHA ideal CVH metrics and ICAS.

Previous studies showed the joint effects of different risk factors on ICAS. Wong et al found that the prevalence of ICAS escalated progressively with an increasing number of risk factors (including hypertension, diabetes, hyperlipidemia and advancing age), increasing from 7.2% for one factor to 29.6% for four in 3,057 asymptomatic high-risk patients who had at least one vascular risk factor for ICAS [24]. Similar results were found in a general population of 590 asymptomatic villagers in China. The prevalence of ICAS increased dramatically from 4.4% among those without any risk factors to 30%–50% among those with at least two risk factors (including hypertension, glycosuria, heart diseases and family history of stroke) [19]. In the AsIA (Barcelona-Asymptomatic Intracranial Atherosclerosis) study, REGICOR (the Framingham Functions were adapted and validated in the Spanish population) and Framingham vascular risk scores were calculated. The prevalence of AsIA was progressively higher with increasing number of risk factors, and the higher the REGICOR scores the greater the prevalence of AsIA [20]. Our previous study also showed the role of Framingham stroke risk model in predicting ICAS [17]. Some studies, including the Kailuan Study, showed significant protective effect of ideal CVH metrics on cardiovascular diseases and stroke [18], [25], [26]. The results from this current study suggested that the ideal CVH metrics may also be inversely related with ICAS, which is a main cause of ischemic stroke [8], [9], in general Chinese populations.

The effect of the ideal CVH metrics may be different between male (p-trend<0.0001) and female (p-trend = 0.0209). Females are more likely to meet more ideal metrics and the number of women who met only one or no ideal metric was relatively small, which may give some reason for different effects between genders. Meanwhile, it is more difficult to observe the TCD signals through temporal bone in older women than in older men [27], which may underestimate the true prevalence of ICAS in women. Furthermore, the difference between genders may be partly due to impacts of sex hormone [28]. Previous studies showed that progression of atherosclerosis was different between males and females [29]. ICAS developed earlier in males, while it progressed faster in females, in which menopause might play a role [22]. Estrogen might enhance NO-mediated relaxation in the intracranial arteries, reduce NADPH-oxidase activity, and give some long-term indirect effects via reduction of cardiovascular risk factors [30], [31], [32]. The relationship between the menstruation status/sex hormone and cerebrovascular diseases needs to be investigated further.

We also examined the relationships between each individual CVH metric and the prevalence of ICAS. In accordance with previous studies [19], [20], [21], [22], [23], [33], we found a significant inverse association between ideal blood pressure or fasting blood glucose and the prevalence of ICAS. Total cholesterol was reported to be a modifiable risk factor for ICAS [34], [35]. One study on 935 Koreans reported that total cholesterol had different effects on ICAS between genders [28]. Hypercholesterolemia was an independent risk factor for ICAS in male participants, but not in female participants, which was consistent with our results. They also provided further analysis in different age groups (<50 years versus ≥50 years), and showed the difference was more significant in the elder group, which suggested that the role of hypercholesterolemia in the development of ICAS may be influenced more by diminishing androgen in males than by reducing sex hormone levels in females [28]. Nevertheless, other studies showed some controversial results and found no significant correlation [21], [22], [33], [36], which might be related to the interaction between hypercholesterolemia and gender [28]. One previous study pointed out that smoking was the most significant independent predictor for intracranial carotid atherosclerosis, and the duration of smoking and hypertension were more powerful than serum lipid level to predict ICAS [37]. Another study found smoking was significantly associated with ICAS in males, not in females [28]. Our study showed similar results. However, Bae HJ et al’s study found no association between smoking and ICAS [22]. This inconsistency may be explained by the difference of age or sex in the selected population, as shown in our subgroup analyses. We found no inverse association between BMI and ICAS, which was consistent with a previous study [24]. Some studies found a U-shaped relationship between BMI and all-cause mortality, and both overweight and underweight were associated with increased mortality [38], [39], [40], which may be one reason for the above result. Further studies considering potential confounders such as occult malignancy, malnutrition, and so on are needed to confirm the true relationship. Lack of comprehensive evaluations on diet or physical activity, we did not find any association between these two metrics and ICAS.

Our study showed healthy behaviors/factors were associated with the prevalence of ICAS, which is quite meaningful in a general population. Some limitations of our study also need to be considered. First, as it was a cross-sectional study, we were not able to evaluate the effect of AHA CVH metrics on the development of intracranial atherosclerosis. We did not know if the risk factors were present before or after the development of ICAS and the real cause-and-effect relationship between the two. However, we did find an inverse relationship between the number of ideal metrics and ICAS. Prospective studies are needed to understand the temporal direction of the observed association between AHA CVH metrics and ICAS risk. As the study is ongoing, we will explore this in the future. Second, the APAC study is not a nationally representative sample and our findings may not be generalized directly to other Chinese populations with different educational, economic and cultural backgrounds.Third, the measurement of diet and physical activity was also not perfect because we did not use any validated dietary and physical activity questionnaires. This may be the reason why there is no significant relationship between diet or physical activity and ICAS found in our study. Fourth, TCD is the only diagnostic tool without confirmation with MRA (Magnetic Resonance Angiography) or other forms of angiography. However, TCD is a widely accepted method for screening intracranial occlusive diseases with a high negative predictive value [41], [42], [43], [44], [45], especially in a general population, and is well accepted by patients as a non-invasive test with a low cost. Future studies will consider the use of other supplementary diagnostic tools to rectify this weakness. We also regarded participants without a good temporal window as non-stenotic, which might underestimate the true prevalence of ICAS in the population, particularly among older participants. Finally, lacking of histopathological information of the lesion responsible for arterial stenosis, we are unable to identify the different etiologies and pathogenesis, for example, atherosclerotic and nonatherosclerotic, such as dissection, moyamoya disease, vasculitis and fibromuscular dysplasia, which may have different risk factors and preventive strategies.

### Conclusion

We observed a clear gradient inverse relationship between the number of ideal CVH metrics and the prevalence of ICAS in a Chinese population, which supports the importance of ideal health behaviors and factors in the prevention of ICAS.

## Supporting Information

Table S1 **Changes in Area under the Receiver-operating Curve (AROC) for Different Metrics.** Basic: age, sex, education, income, family history of stroke; BP: blood pressure; FBG: fasting blood glucose; TC: total cholesterol; BMI: body mass index; PA: physical activity
(DOCX)

Table S2 Odds Ratios (ORs) with 95% CI for ICAS according to Each Individual Health Metric*. CI: confidence interval; ICAS: intracranial artery stenosis; BMI: body mass index. *The following potential confounders were adjusted for each OR: sex, age (year), education, average monthly income of each family member, family history of stroke, and the other six cardiovascular health metrics.
(DOCX)
