# Supplementary material for: Ideal Cardiovascular Health Metrics on the Prevalence of Asymptomatic Intracranial Artery Stenosis: A Cross-Sectional Study
Source: PLoS One. 2013 Mar 12;8(3):e58923. doi: 10.1371/journal.pone.0058923 (PMC3595221; doi:10.1371/journal.pone.0058923)
Supplement: Table S1 — Changes in Area under the Receiver-operating Curve (AROC) for Different Metrics. Basic: age, sex, education, income, family history of stroke; BP: blood pressure; FBG: fasting blood glucose; TC: total cholesterol; BMI: body mass index; PA: physical activity (DOCX) [file pone.0058923.s001.docx]

**Table S1. Changes in Area under the Receiver-operating Curve (AROC) for Different Metrics**

| Variables used in the analysis | | | | | | | | AROC |
| --- | --- | --- | --- | --- | --- | --- | --- | --- |
| Basic | BP | FBG | smoking | TC | diet | BMI | PA |  |
| **+** |  |  |  |  |  |  |  | 0.656 |
| **+** | **+** |  |  |  |  |  |  | 0.673 |
| **+** | **+** | **+** |  |  |  |  |  | 0.683 |
| **+** | **+** | **+** | **+** |  |  |  |  | 0.686 |
| **+** | **+** | **+** | **+** | **+** |  |  |  | 0.689 |
| **+** | **+** | **+** | **+** | **+** | **+** |  |  | 0.691 |
| **+** | **+** | **+** | **+** | **+** | **+** | **+** |  | 0.691 |
| **+** | **+** | **+** | **+** | **+** | **+** | **+** | **+** | 0.691 |

Basic: age, sex, education, income, family history of stroke

BP: blood pressure

FBG: fasting blood glucose

TC: total cholesterol

BMI: body mass index

PA: physical activity
