# Supplementary material for: Ideal Cardiovascular Health Metrics on the Prevalence of Asymptomatic Intracranial Artery Stenosis: A Cross-Sectional Study
Source: PLoS One. 2013 Mar 12;8(3):e58923. doi: 10.1371/journal.pone.0058923 (PMC3595221; doi:10.1371/journal.pone.0058923)
Supplement: Table S2 — Odds Ratios (ORs) with 95% CI for ICAS according to Each Individual Health Metric*. CI: confidence interval; ICAS: intracranial artery stenosis; BMI: body mass index. *The following potential confounders were adjusted for each OR: sex, age (year), education, average monthly income of each family member, family history of stroke, and the other six cardiovascular health metrics. (DOCX) [file pone.0058923.s002.docx]

**Table S2. Odds Ratios (ORs) with 95% CI for ICAS according to Each Individual Health Metric** *

| Metrics | Total | Gender , OR（95% CI） | | Age, OR（95% CI） | |
| --- | --- | --- | --- | --- | --- |
|  |  | Male | Female | 40-60(y) | ≥60(y) |
| Smoking |  |  |  |  |  |
| Ideal | 0.78(0.62-0.98) | 0.64(0.50-0.82) | 2.07 (0.62-6.98) | 0.78(0.56-1.10) | 0.64(0.46-0.88) |
| Intermediate | 1.22(0.88-1.69) | 1.003(0.71-1.42) | 9.83(1.36-70.98) | 1.05(0.58-1.91) | 1.07(0.70-1.63) |
| Poor | 1 | 1 | 1 | 1 | 1 |
| P value | 0.0125 | 0.0009 | 0.0745 | 0.3385 | 0.0049 |
| BMI (kg/m2) |  |  |  |  |  |
| Ideal | 1.34(0.94-1.92) | 1.13(0.70-1.82) | 1.54(0.88-2.69) | 1.38(0.86-2.23) | 1.30(0.75-2.27) |
| Intermediate | 1.35(0.94-0.93) | 1.32(0.82-2.12) | 1.29(0.73-2.26) | 1.39(0.86-2.24) | 1.31(0.75-2.29) |
| Non-ideal | 1 | 1 | 1 | 1 | 1 |
| P value | 0.2537 | 0.2756 | 0.2157 | 0.3907 | 0.6303 |
| Physical activity |  |  |  |  |  |
| Ideal | 0.97(0.80-1.17) | 0.93(0.73-1.20) | 1.02(0.74-1.40) | 0.97(0.74-1.26) | 0.94(0.69-1.27) |
| Intermediate | 0.96(0.77-1.19) | 0.91(0.68-1.22) | 0.99(0.71-1.38) | 0.84(0.63-1.11) | 1.12(0.78-1.61) |
| Non-ideal | 1 | 1 | 1 | 1 | 1 |
| P value | 0.9042 | 0.7858 | 0.9830 | 0.4617 | 0.5733 |
| Diet |  |  |  |  |  |
| Ideal | 1.24(0.96-1.61) | 1.32 (0.95-1.84) | 1.23(0.80-1.90) | 1.26(0.89-1.77) | 1.31(0.88-1.95) |
| Intermediate | 1.04(0.83-1.29) | 1.05(0.80-1.37) | 1.04(0.71-1.53) | 1.02(0.76-1.37) | 1.07(0.77-1.50) |
| Non-ideal | 1 | 1 | 1 | 1 | 1 |
| P value | 0.1589 | 0.1862 | 0.5065 | 0.2785 | 0.3413 |
| Total cholesterol |  |  |  |  |  |
| Ideal | 0.80(0.62-1.02) | 0.64(0.47-0.88) | 0.997(0.65-1.52) | 0.80 (0.57-1.14) | 0.68(0.47-0.98) |
| Intermediate | 1.09(0.84-1.41) | 1.09(0.78-1.50) | 1.07(0.70-1.66) | 1.16(0.81-1.67) | 0.93(0.64-1.36) |
| Non-ideal | 1 | 1 | 1 | 1 | 1 |
| P value | 0.0024 | <0.0001 | 0.8800 | 0.0109 | 0.0333 |
| Blood pressure |  |  |  |  |  |
| Ideal | 0.47(0.35-0.64) | 0.32(0.18-0.54) | 0.47(0.32-0.69) | 0.45(0.32-0.63) | 0.35(0.18-0.66) |
| Intermediate | 0.89(0.74-1.06) | 0.93(0.74-1.17) | 0.87(0.65-1.17) | 0.74(0.58-0.95) | 1.16(0.89-1.51) |
| Non-ideal | 1 | 1 | 1 | 1 | 1 |
| P value | <0.0001 | 0.0001 | 0.0007 | <0.0001 | 0.0017 |
| Fasting blood glucose |  |  |  |  |  |
| Ideal | 0.48(0.38-0.61) | 0.47(0.35-0.63) | 0.47(0.30-0.72) | 0.46(0.32-0.64) | 0.47(0.33-0.66) |
| Intermediate | 0.68(0.53-0.89) | 0.68(0.49-0.93) | 0.72(0.45-1.14) | 0.59(0.41-0.85) | 0.82(0.56-1.20) |
| Non-ideal | 1 | 1 | 1 | 1 | 1 |
| P value | <0.0001 | <0.0001 | 0.0005 | <0.0001 | <0.0001 |
| AROC | 0.694 | 0.740 | 0.652 | 0.646 | 0.664 |

CI: confidence interval; ICAS: intracranial artery stenosis; BMI: body mass index

*The following potential confounders were adjusted for each OR: sex, age (year), education, average monthly income of each family member, family history of stroke, and the other six cardiovascular health metrics.
